# Supplementary material for: A gigabyte interpreted seismic dataset for automatic fault recognition
Source: Data Brief. 2021 Jun 12;37:107219. doi: 10.1016/j.dib.2021.107219 (PMC8220338; doi:10.1016/j.dib.2021.107219)
Supplement: Supplementary Data S1 — Supplementary Raw Research Data. This is open data under the CC BY license http://creativecommons.org/licenses/by/4.0/ [file mmc1.pdf]

Data in Brief (2021)

Contents lists available at ScienceDirect

Data in Brief

journal homepage: www.elsevier.com/locate/dib

Data Article

A Gigabyte Interpreted Seismic Dataset for Automatic Fault Recognition

Yu An[a,*], Jiulin Guo[b], Qing Ye[c], Conrad Childs[d], John Walsh[d,e], Ruihai Dong[a]

[a]*The Insight Centre for Data Analytics, School of Computer Science, University College Dublin, Dublin, Ireland*
[b]*C&C Reservoirs, Brunel House, Reading, United Kingdom*
[c]*Key Laboratory of Tectonics and Petroleum Resources, Ministry of Education, China University of Geosciences, Wuhan 430074, China*
[d]*Fault Analysis Group, School of Earth Sciences, University College Dublin, Dublin, Ireland*
[e]*iCRAG (Irish Centre for Research in Applied Geosciences)*

ARTICLE INFO

Keywords:

Fault Recognition
Seismic Interpretation
Computer Vision
Image Processing

ABSTRACT

The lack of large-scale open-source expert-labelled seismic datasets is one of the barriers to applying today's AI techniques to automatic fault recognition tasks. The dataset present in this article consists of a large number of processed seismic images and their corresponding fault annotations. The processed seismic images, which are originally from a seismic survey called Thebe Gas Field in the Exmouth Plateau of the Carnarvan Basin on the NW shelf of Australia, are represented in Python Numpy format, which can be easily adopted by various AI models and will facilitate cooperation with researchers in the field of computer science. The corresponding fault annotations were firstly manually labelled by expert interpreters of faults from seismic data in order to investigate the structural style and associated evolution of the basin. Then the fault interpretation and seismic survey are processed and collected using Petrel software and Python programs separately. This dataset can help to train, validate, and evaluate the performance of different automatic fault recognition workflow.

http://dx.doi.org/10.1016/j.dib.xxxx.xx.xxx

2352-3409/© 2021 The Author(s). Published by Elsevier Inc.

Specifications Table

Subject	Computers in Earth Sciences
Specific subject area	Computer vision for automatic geological fault recognition
Type of data	Image stored in NumPy [1] array format
How data were acquired	Data were first interpreted manually using professional software Petrel. Then, processed and collected by Petrel software and Python programs.
Data format	Raw and Analysed
Parameters for data collection	Only faults with vertical displacements greater than 20 m within a particular area of interest and depth range (ca 2km to 4km) are considered.
Description of data collection	Data were collected using custom workflow function in the professional software called Petrel and post-processed using Python programs
Data source location	Institution: University College Dublin<br>City/Town/Region: Dublin<br>Country: Ireland<br><br>Primary data source:<br>Institution: Geoscience Australia, National Offshore Petroleum Titles Administrator<br>City/Town/Region: Carnarvan Basin<br>Country: Australia<br>Latitude and longitude: 19°25'24.49"S, 113°5'19.44"E
Data accessibility	Repository name: Harvard Dataverse<br>Data identification number: https://doi.org/10.7910/DVN/YBYGBK<br>Direct URL to data:<br>https://dataverse.harvard.edu/dataset.xhtml?persistentId=doi:10.7910/DVN/YBYGBK [2]
Related research article	Y. An, J. Guo, Q. Ye, C. Childs, J. Walsh, R. Dong, Deep convolutional neural network for automatic fault recognition from 3d seismic datasets, Computers & Geosciences 153 (2021) 104776. doi: https://doi.org/10.1016/j.cageo.2021.104776 [3]<br>Y. An, Q. Ye, J. Guo, R. Dong, Overlap training to mitigate inconsistencies caused by image tiling in cnns, in: M. Bramer, R. Ellis (Eds.), Artificial Intelligence XXXVII, Springer International Publishing, Cham, 2020, pp.35–48. doi: 10.1007/978-3-030-63799-63. [4]

Value of the Data

- Expert assessment and synthetic datasets are often used to evaluate different fault recognition algorithms due to the lack of large-scale public interpreted seismic datasets. With the help of this dataset, researchers can systematically evaluate the performance of different artificial intelligence algorithms.
- This dataset will be useful to researchers who are developing and testing algorithms for automatic fault recognition.
- This dataset can be used for comparing the performance of different (2D and 3D) automatic fault recognition algorithms.
- Alternatively, the data collection method introduced in this paper will be beneficial for researchers to generate similar datasets.
- The data set is processed in Python NumPy format instead of a specific file format used in the field of earth sciences, facilitating collaboration with researchers in computer science and the use of modern artificial intelligence methods.

*Corresponding author
e-mail: yu.an@insight-centre.org (Yu An)

Data Description

Fault recognition is the process of identifying and annotate planer fractures from the earth's crust, which can also be seen as a process of annotating certain type of discontinuous from seismic images (i.e. acoustic reflection imaging of underground rock structures). This process is still dominated by manual interpretation, and it takes several weeks to several months, depending on data quality and experience. The exponentially increasing amount of data and the need for fast and accurate fault recognition have led many researchers to pay attention to the topic of automatic fault recognition. In this case, a large-scale open-source expert-labelled dataset can effectively supplement or even replace the expert assessment, to systematically analyse and compare the performance of different automatic fault recognition algorithms. To this end, we present a gigabyte interpreted seismic dataset for automatic fault recognition, which can be accessed through this link `https://doi.org/10.7910/DVN/YBYGBK`.

The provided dataset repository contains four main folders (i.e. data, code, docs, and license) and 42 files in total. The data folder contains three sub-folders (i.e. raw, seis, fault), which stores one raw fault annotation file, 18 processed seismic data and 18 corresponding processed fault annotations. The raw fault annotation file is directly exported from the professional fault interpretation software Petrel, and stored in the domain standard file format: ESXI also called ASCII. The remaining 18 pairs of processed data files are stored in the Python NumPy (i.e. .npy) file format so that they can be directly used in current machine learning algorithms. Especially nowadays, the majority of deep learning algorithms are compiled with python language. The code folder contains two code files: readSGY.ipynb and preprocessimages.ipynb. Seismic data is usually stored in SEGY/SGY file format. The readSGY.ipynb file describes in detail the process of reading the SEGY file and converting it to NumPy file format. The preprocessimages.ipynb file illustrate the process of converting image format annotations like Fig. 1(c) to NumPy files like Fig. 1(d). The documentation folder contains a PDF file that provides step-by-step instructions for generating fault annotation screenshots from ASCII fault stick files using a custom Petrel workflow. Finally, the license folder contains two license file: Creative Commons Attribution 4_0 International CC BY 4_0.pdf and Summary Creative Commons Attribution 4.pdf.

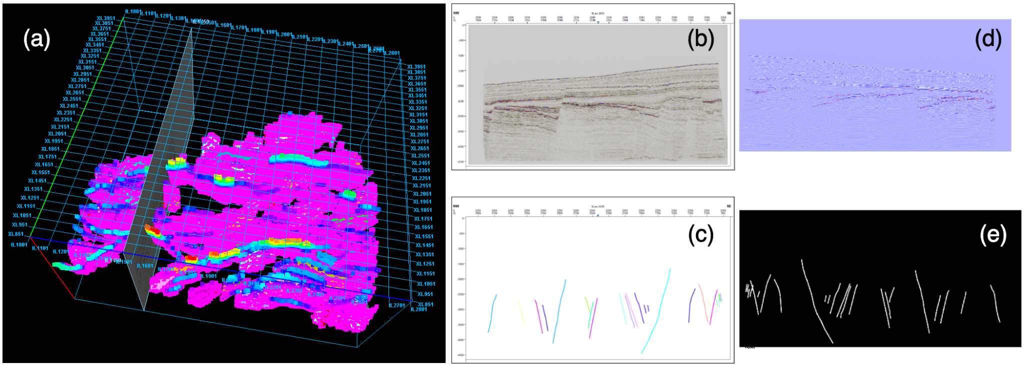

Fig. 1: Illustration of the dataset. (a) 3D view. (b) IL2005 seismic data screenshot. (c) IL2005 fault annotations screenshot (d) IL2005 processed seismic image. Originally one channel image, illustrated in colour for better visualization. (e) IL2005 processed fault annotations

Fig. 1 provides a general illustration of the described dataset. Fig. 1(a) is a 3D view of the raw fault annotations, derived from raw seismic data of roughly the same non-rectangular shape.

The raw seismic data/survey, which is called Thebe Gas Field, is located in the Exmouth Plateau of the Carnarvan Basin on the NW shelf of Australia. Fig. 1(b) and (c) are 2D cross-section views of seismic data and fault annotations collected using Petrel workflow. Fig. 1(d) and (e) are corresponding processed seismic data and fault annotations with the size of 1803 [crossline] × 3174 [inline] × 1537 [sample]. The fault annotations are expressed in binary format, where true means a fault passes through the voxel. Otherwise, no fault passes.

Fig. 2 illustrates the data preparation process, during which SEGY format seismic data and IESX format also called ASCII format fault sticks are processed to two 3D rectangular NumPy volumes. Details are explained in the next section Experimental Design, Materials and Methods.

The seismic data and expert annotations are divided into a training set, a validation set, and a test set and stored in three folders (train, val, test) to effectively train and compare different machine learning algorithms. The first 900 cross-sections with size of 900 [crossline] × 3174 [inline] × 1537 [sample] are divided into the training set for adjusting algorithm parameters. Similarly, the next 200 cross-sections with the size of 200 [crossline] × 3174 [inline] × 1537 [sample] are divided into the validation set to test the performance of the model during the training process, and the last 703 cross-sections with the size of 703 [crossline] × 3174 [inline] × 1537 [sample] are divided into the test set to evaluate the performance of different algorithms objectively. Due to the large size of the dataset, we sequentially divide each set into smaller files. Each file contains 100 cross-sections, with a size of 3.9G (seis*.npy) or 465.2M (fault*.npy). Except for the last pair of test set files, they all contain 103 cross-sections with a size of 4.02G (seistest7.npy) or 479.2M (faulttest7.npy).

Experimental Design, Materials and Methods

As mentioned in many related works, [5, 6, 7, 8], a common challenge for a comprehensive evaluation of automatic fault recognition performance is the lack of large-scale open-source interpreted seismic datasets. Therefore, two countermeasures have emerged. One countermeasure is to interpret a few cross-sections (e.g. three cross-sections with a size of 484 × 151 pixels [8]) to adjust the algorithm parameters and then provide a few visual examples for expert assessment. Another method is to use the synthetic dataset to train the algorithm parameters, and then still use expert evaluation to illustrate the performance roughly [5, 6]. Neither of these two countermeasures can comprehensively and objectively evaluate the performance of different fault recognition algorithms on real-world seismic datasets. We pay attention to this issue and cooperate with geologists to generate a large-scale expert-labelled fault recognition dataset.

The dataset preparation process, as shown in Fig. 2 includes three steps: expert annotation, data collection and data pre-processing.

Expert annotation

Geological fault interpretation is a complicated and time-consuming process. Depending on different research purposes, the area of interest will be different. In this dataset, experts (from Fault Analysis Group, University College Dublin) only annotate geological faults with vertical displacements greater than 20 m in specific areas of interest and depth ranges (about 2km to 4km). The interpretation process is performed on a domain-specific software called Petrel.

Experts use polylines to annotate specific discontinuous patterns (i.e. faults) on discrete 2D cross-section views (i.e. the process of annotate Fig. 1(b) to Fig. 1(c)). These polylines are stored as 3D points using ASCII file format, which can be automatically connected into surfaces through professional software e.g. Petrel.

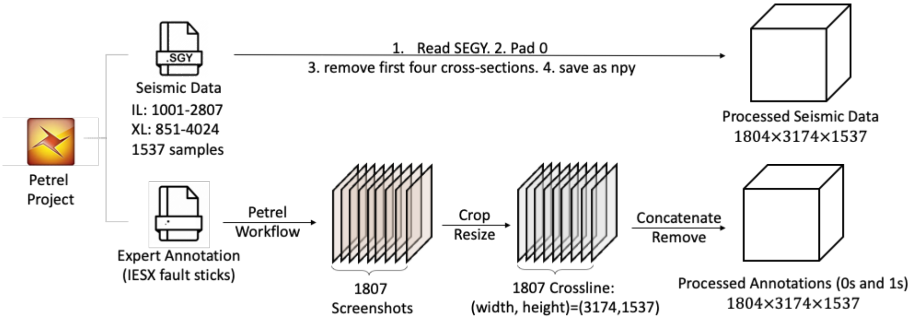

Fig. 2: The procedure for converting Petrel projects into processed NumPy dataset. Modified from [3]

Data collection

A Petrel project is created after the annotation process. Then, we designed a customised Petrel workflow to generate an as large as possible fault recognition dataset, that is, generate corresponding fault annotation for each of the cross-sections. Petrel's workflow is similar to programs, which can customise to repeat certain build-in operations multiple times. In this workflow, screenshots of paired seismic data and fault annotations are taken for each cross-section. In total, 1807 screenshot pairs are token for 1807 cross-sections.

Data pre-processing

The data pre-processing process can be divided into two parts. One for seismic data and the other one for fault annotations' pre-processing. The seismic data used in this paper is stored in the standard SGY file format, can be only read using specific methods. To maintain seismic details and minimise data migration loss, we use python programs to directly read the data. Though the seismic data has three coordinates, with inline (IL or X) number range from 1001 to 2807, crossline (XL or Y) number range from 851 to 4024 and 1537 samples (depth or Z), it is not a rectangular shape, see Fig. 1(a). Zeros are used to pad the seismic data to a rectangular shape with the size of 1807 [crossline] $\times$ 3174 [inline] $\times$ 1537 [sample]. We removed the first four of 1807 cross-sections as we observed that the experts did not provide any annotations on them. Finally, the processed seismic data are stored as a NumPy volume with the size of 1804 [crossline] $\times$ 3174 [inline] $\times$ 1537 [sample].

For fault annotation, the data collection process will produce 1807 coloured screenshots with the size of screen display resolution and also contain axis information, as shown in Fig. 1(c). A python program (i.e. preprocessimages.ipynb) was designed to read and process these screenshots. Since using different colours is only for 3D fault surface construction and visualization, we ignore the colour difference, read them into binary (i.e. gray-scale) format. To provide accurate seismic and annotation pairs, we first cut the axis boundary according to the seismic screenshot boundary, as shown in Figure 1(b). Then, we scale them to the size of the seismic cross-section's size, which is 3174 $\times$ 1573 pixels. Similarly, we also removed the first four of 1807 cross-sections. Moreover, we flip them horizontally to ensure consistency with the original seismic dataset direction. Finally, we concatenate them and saved them to a NumPy volume with the same shape as the processed seismic data.

Considering that we created this dataset mainly to train and evaluate automatic fault recognition algorithms, we follow the machine learning dataset segmentation principle and divide the

dataset into a training set, a validation set and a test set, as described in section Data Description. We hope to build a public leader board based on this dataset. It will help relevant researchers to test and compare the performance of different machine learning algorithms.

Ethics Statement

This dataset does not include any studies in humans and animals.

CRediT author statement

Yu An: Writing- Original draft preparation, Conceptualization, Methodology, Software **Jiulin Guo**: Conceptualization, Methodology. **Qing Ye**: Interpretation, Conceptualization, Methodology. **Conrad Childs**: Supervision. **John Walsh**: Supervision. **Ruihai Dong**: Supervision.

Acknowledgments

Thanks to Australia National Offshore Petroleum Information Management System for providing the original Thebe dataset. Thanks to Niall Reenan, Dr Marcus Carneiro, Dr Eoin Dunlevy and Luo Luo from UCD for providing help on datasets and Petrel software. This publication has emanated from research supported in part by a grant from Science Foundation Ireland under Grant number [SFI/12/RC/2289_P2, SFI/13/RC/2092]. For the purpose of Open Access, the author has applied a CC BY public copyright licence to any Author Accepted Manuscript version arising from this submission. This work was also supported by the European Regional Development Fund; Beijing-Dublin International College Fund.

Declaration of Competing Interest

The seismic data in our dataset is a reproduced version of survey data by Geoscience Australia under license: Creative Commons Attribution 4.0 International CC BY 4.0.

References

[1] C. R. Harris, K. J. Millman, S. J. van der Walt, R. Gommers, P. Virtanen, D. Cournapeau, E. Wieser, J. Taylor, S. Berg, N. J. Smith, R. Kern, M. Picus, S. Hoyer, M. H. van Kerkwijk, M. Brett, A. Haldane, J. F. del R'ıo, M. Wiebe, P. Peterson, P. G'erard-Marchant, K. Sheppard, T. Reddy, W. Weckesser, H. Abbasi, C. Gohlke, T. E. Oliphant, Array programming with NumPy, Nature 585 (2020) 357–362. doi: 10.1038/s41586-020-2649-2.

[2] [Dataset] Y. An, J. Guo, Q. Ye, C. Childs, J. Walsh, R. Dong, A Gigabyte Interpreted Seismic Dataset for Automatic Fault Recognition. Harvard Dataverse, V2, 2021. doi: 10.7910/DVN/YBYGBK.

[3] Y. An, J. Guo, Q. Ye, C. Childs, J. Walsh, R. Dong, Deep convolutional neural network for automatic fault recognition from 3d seismic datasets, Computers & Geosciences 153 (2021) 104776. doi: https://doi.org/10.1016/j.cageo.2021.104776.

[4] Y. An, Q. Ye, J. Guo, R. Dong, Overlap training to mitigate inconsistencies caused by image tiling in cnns, in: M. Bramer, R. Ellis (Eds.), Artificial Intelligence XXXVII, Springer International Publishing, Cham, 2020, pp. 35–48. doi: 10.1007/978-3-030-63799-6_3.

[5] X. Wu, L. Liang, Y. Shi, S. Fomel, Faultseg3d: Using synthetic data sets to train an end-to-end convolutional neural network for 3d seismic fault segmentation, Geophysics 84 (2019) IM35–IM45. doi: 10.1190/geo2018-0646.1.

[6] X. Wu, Z. Geng, Y. Shi, N. Pham, S. Fomel, Building realistic structure models to train convolutional neural networks for seismic structural interpretation, in: SEG Technical Program Expanded Abstracts 2019, Society of Exploration Geophysicists, 2019, pp. 4745–4750. doi: 10.1190/segam2019-3214282.1.

[7] A. Cunha, A. Pochet, H. Lopes, M. Gattass, Seismic fault detection in real data using transfer learning from a convolutional neural network pre-trained with synthetic seismic data, Computers & Geosciences 135 (2020) 104344. doi: 10.1016/j.cageo.2019.104344.
[8] H. Di, Z. Wang, G. AlRegib, Seismic fault detection from post-stack amplitude by convolutional neural networks, in: 80th EAGE Conference and Exhibition 2018, European Association of Geoscientists & Engineers, 2018, pp. 1–5. doi: 10.3997/2214-4609.201800733.
